# Steady-state versus burst lasing techniques for thulium fiber laser

**DOI:** 10.1007/s00345-024-05102-1

**Published:** 2024-08-19

**Authors:** Alba Sierra, Catalina Solano, Mariela Corrales, Eugenio Ventimiglia, Frederic Panthier, Jia-Lun Kwok, Marie Chicaud, Etienne Xavier Keller, Olivier Traxer

**Affiliations:** 1 Urology Department, Hospital Clínic de Barcelona, Universitat de Barcelona, Villarroel 170, 08036 Barcelona, Spain; 2 Sorbonne University GRC Urolithiasis No. 20 Tenon Hospital, 75020 Paris, France; 3 Department of Urology AP-HP, Tenon Hospital, Sorbonne University, 75020 Paris, France; 4 Progressive Endourological Association for Research and Leading Solutions (PEARLS Group), Paris, France; 5 Uroclin, Medellín, Colombia; 6 Division of Experimental Oncology/Unit of Urology, URI, IRCCS Ospedale San Raffaele, Università Vita-Salute San Raffaele, Milan, Italy; 7 Departament of Urology, University Hospital Zürich, Zurich, Switzerland; 8 Departament of Urology, Tan Tock Seng Hospital, Singapore, Singapore; 9 Department of Urology, Limoges University Hospital, 2 Avenue Martin Luther King, 87000 Limoges, France; 10 University Hospital Zurich, University of Zurich, Zurich, Switzerland

**Keywords:** Thulium fiber laser, Urinary stones, Laser settings, Laser efficacy, Thermal damage, Burst, Continuous

## Abstract

**Objective:**

To evaluate the stone ablation rate and direct thermal damage from thulium fiber laser (TFL) lithotripsy using continuous (C) and burst (B) lasing techniques on an in vitro ureteral model.

**Methods:**

The TFL Drive (Coloplast, Humlebaek, Denmark) was used in an in vitro saline-submerged ureteral model. Ten participants, including five junior and five experienced urologists, conducted the experimental setup with 7 different settings comparing two lasing techniques: steady-state lasing (0.5 J/10 Hz = 5W for 300 s and 0.5 J/20 Hz = 10W for 150 s) and burst, intermittent 5 s on/off lasing (0.5 J/20 Hz, 0.5 J/30 Hz, 0.5 J/60 Hz, 0.1 J/200 Hz, and 0.05 J/400 Hz) with a target cumulative energy of 1500 J using cubic 125 mm^3^ phantom BegoStonesTM. Ureteral damage was graded 1–3 based on the severity of burns and holes observed on the surface of the ureteral model.

**Results:**

The were no significant differences in stone ablation mass neither between C and B lasing techniques, nor between expertise levels. At C lasing technique had only mild ureteral lesions with no significant differences between expertise levels (p: 0.97) or laser settings (p: 0.71). At B lasing technique, different types of thermal lesions were found with no expertise (p: 0.11) or setting (p: 0.83) differences. However, B laser setting had higher grade direct thermal lesions than C (p: 0.048).

**Conclusion:**

Regarding efficacy, C and B lasing techniques achieve comparable stone ablation rates. Safety-wise, B lasing mode showed higher grade of direct thermal lesions. These results should be further investigated to verify which of the lasing mode is the safest in vivo. Until then and unless proven otherwise, a C mode with low frequency should be recommended to avoid ureteral wall lesions.

## Introduction

In recent years, the thulium fiber laser (TFL) has emerged as a promising alternative to the holmium YAG (Ho:YAG) laser for lithotripsy in urology, potentially offering safer and more effective treatment [1–4].

Studies comparing TFL and Ho:YAG lasers have shown TFL's superiority, supported by prospective randomized studies and case series, despite ongoing safety debates [5–7]. However, safety has become a topic of debate, with a recent upward trend in intraoperative complications and the latest FDA “Class 2 Device Recall” to Olympus for thermal injury following dusting and fragmenting treatment of ureteral stones when users exceeded the 20W standard presets [8–10]. While pulse modulation devices seem to avoid stone push-up, reducing ureteral lesions, postoperative complications, and ureteral strictures [11]. Ureteral strictures can result from the thermal effect of lasers, exacerbated by high-power settings leading to elevated temperatures and tissue damage [12, 13].

High-power and high-frequency settings can cause a "snowstorm" effect, obstructing vision and necessitating intermittent laser activation, known as burst lasing, to manage thermal effects and improve visibility [14]. Burst lasing intermittently stops laser emission, allowing time for irrigation to cool the medium. On the other hand, steady-state lasing maintains a continuous beam for optimal visibility and effective stone dusting, typically at low frequencies [15].

The main safety concern lies in uncontrolled lasing during burst mode, prompting a study to evaluate stone ablation rate and thermal injury using burst and steady-state techniques in vitro. The study aims to address this knowledge gap by meticulously assessing the effects of different lasing techniques on safety and efficacy.

## Materials and methods

### Experimental setup

**Fig. 1:**
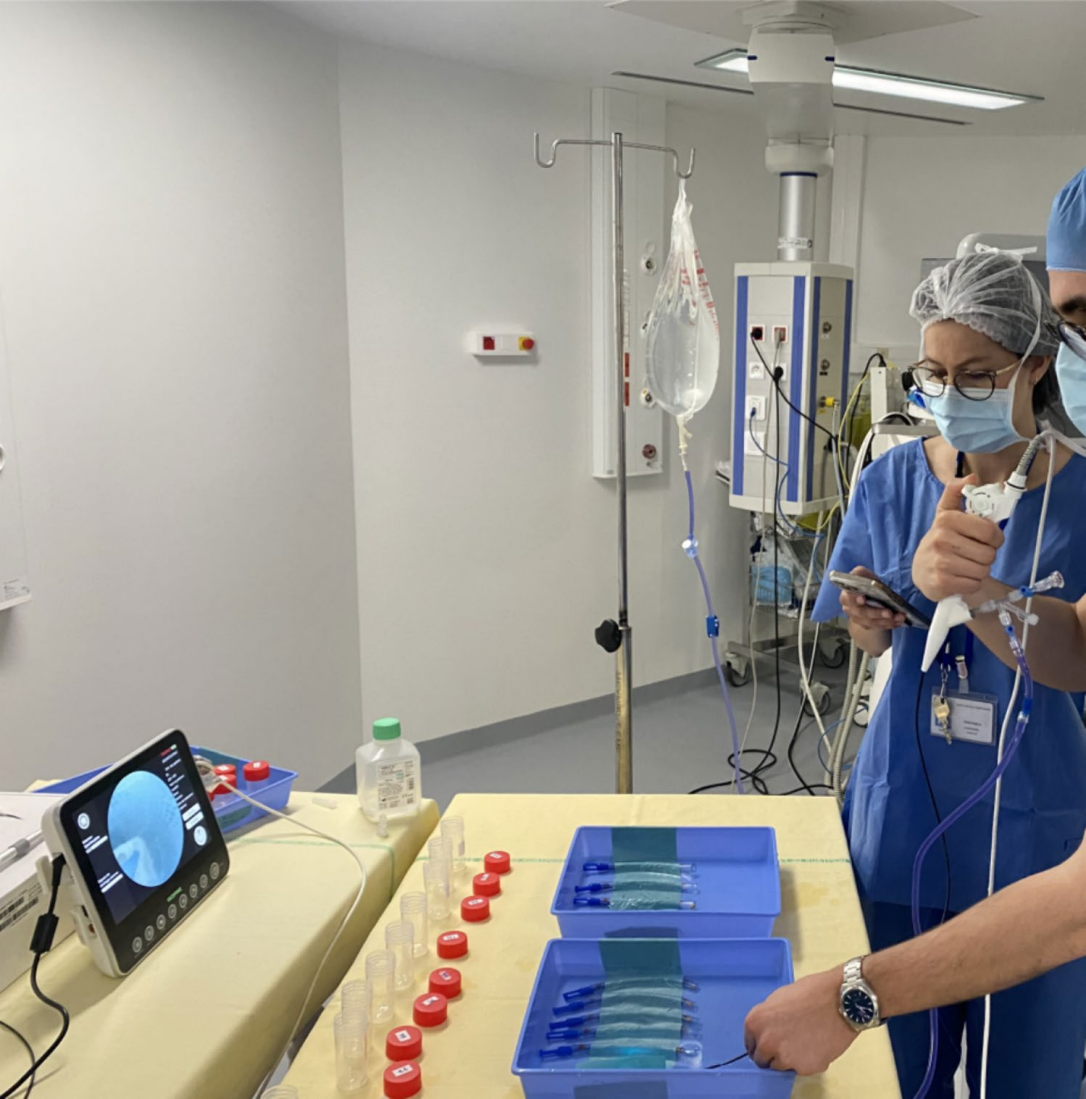
Experimental set up. Six polymer tubes, 17 cm length, closed on one side, 7 mm diameter, with an opaque tape on a tray with saline, were used as a ureteral model. A Single-use Digital Flexible Ureteroscope (PU2022A, 7.5F, Pusen, Zhuhai, China) was used for endoscopic vision. Irrigation was ensured by a combination of a gravity irrigation at 40 cmH2O above the saline tray and a hand-assisted irrigation system. An timer was placed next to the participant to indicate the 5 s on/off burst lasering

We employed an in vitro ureteral model submerged in a saline solution as reported before (Fig. 1) [13]. Our trials were conducted using a Single-use Digital Flexible Ureteroscope (PU2022A, 7.5Fh, Pusen, Zhuhai, China). Adequate irrigation was maintained by combining gravity irrigation at 40 cmH2O above the saline tray (Fig. 1).

### Stone phantoms

3D-printed cast were used for cubic 125 mm^3^ BegoStone™ phantoms to simulate ureteral stones by using a "powder to water" ratio of 15:3, as described in prior in vitro studies [13, 16, 17]. After fabrication, each stone phantom underwent a 48-h drying period at ambient temperature, and its weight was precisely measured using a digital balance with a 0.001 accuracy.

### Laser system

We used the TFL Drive (60W, Coloplast, Humlebaek, Denmark), together with 200 µm core diameter laser fiber. Target cumulative energy was settled at 1.5 kJ for each trial.

Laser on time (LOT) was previously calculated for each trial according to the laser settings.

$${\text{Laser}}\;{\text{ on }}\;{\text{time}}\;{ }\left( {{\text{LOT}}} \right)\;\left( {\text{s}} \right) = \frac{{\left[ {{\text{Total }}\;{\text{cumulative}}\;{\text{ energy}}\; \left( {1500\;{\text{J}}} \right)} \right]}}{{\left[ {Energy \;\left( {\text{J}} \right)*Frequency \;\left( {{\text{Hz}}} \right)} \right]}}$$

- Continuous lasing mode (C):

“Steady-state lasing” or continuous lasing mode involves uninterrupted activation of the laser pedal from the beginning of lithotripsy. The laser is activated without interruptions and the participant is unable to stop the laser emission until the assistant signals that the target cumulative energy has been achieved; so, LOT and treatment time (TT) were the same.

- Burst lasing mode (B):

Burst lasing mode involves emitting the laser energy in intermittent bursts with a specific on/off cycle. In our study, we established a pattern of 5 s “on” and 5 s “off”, which corresponds to 50% laser active time. A timer with an alarm guides the participant when to activate and deactivate the laser pedal (Fig. 1).

The laser settings for the steady-state and burst lasing modes are detailed in Table 1, all with a short pulse width according to the manufacturer's laser console settings.

**Table 1:** Experimental laser settings

	Energy (J)	Frequency (Hz)	Watts (W)	Laser on time (s)	Treatment time (s)	Laser active time (%)	Total delivered energy (KJ)
C	0.5	10	5	300		100	1.5
	0.5	20	10	150		100	1.5
B	0.5	20	10	150	300	50	1.5
	0.5	30	15	100	200	50	1.5
	0.5	60	30	50	100	50	1.5
	0.1	200	20	75	150	50	1.5
	0.05	400	20	75	150	50	1.5

Two types of laser activation modes were compared: continuous mode where the lasing was not interrupted until the target cumulative energy was reached (1500 J) and the burst mode, where the laser was intermittently activated for on/off periods of 5 s

*C* continuous, *B* burst

### Participants

A total of ten participants were included in the study, divided into two groups: five trainee, less than 4 years of training in urology, and five fellows/experienced urologists. Each participant conducted the experimental setup one with seven different settings, which consisted of two C lithotripsy sessions and five B lithotripsy sessions. They were instructed to fragment the stone using the selected settings and remain standing. They were only aware that in the first part, they needed to continuously press the pedal for lithotripsy, and in the second part, they would follow our instructions for intermittent pedal use, with us indicating when to start and stop pressing the pedal. Each session was concluded upon reaching a delivered energy of 1.5 kJ.

#### Stone ablation mass and ureteral damage assessment

Laser parameters, including laser settings, total energy (in joules), and LOT (in seconds) and TT (in seconds) and laser active time, were meticulously recorded for each lithotripsy session. After the procedure, stone fragments were labelled and dried at room temperature (21 °C) and were weighted again after the lithotripsy treatment. We have a control stone submerged into the saline tray without lithotripsy treatment during the trial, and we stored it in similar conditions, when its weight was the same as before the experiment, we assumed that the rest were dried too.

To assess the efficacy of the laser settings, the stone ablated mass was calculated by dividing the weight difference before and after the lithotripsy treatment [(artificial stone weight before the experiment (mg) − artificial stone weight dried after lithotripsy (mg)] with Artificial Stone Weight before the experiment (mg) and multiplying by 100 [18].

**Fig. 2:**
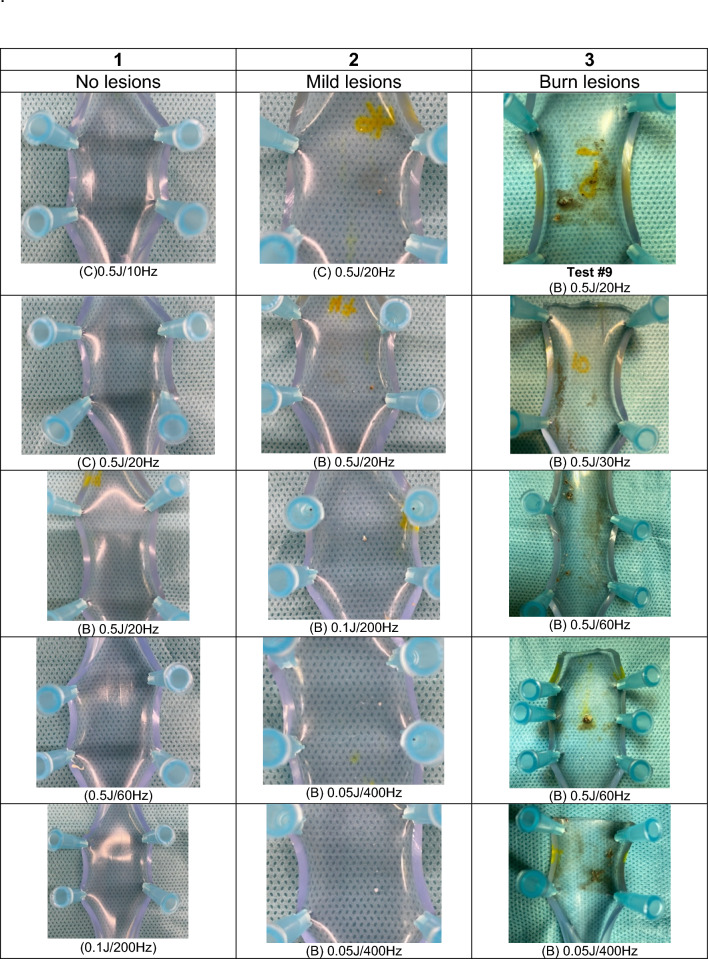
Ureteral model damage classification. Score 1–3 according to the absence of lesions, the presence of impacts/holes and the presence of burns observed on the surface of the ureteral model

Ureteral models used in the experiments were collected, labelled, and bivalved for damage. To assess the thermal lesions, a scale was established according to the findings in the surface of the ureteral model: the absence of lesions, the presence of impacts/holes and the presence of burns, ranging from 1 to 3 respectively (Fig. 2). If there were different types of injuries, the highest grade was prioritized for classification. Plastic tubes were classified blinded according to the thermal impacts score by consensus between two authors (AS, MC).

### Statistical analysis

To analyse the data collected, we employed SPSS v25 software (IBM Corp. Released 2017. IBM SPSS Statistics for Windows, Version 25.0. Armonk, NY: IBM Corp.). Ablation rates for different laser settings and equipment configurations were recorded and subjected to statistical analysis. Ablation rate was assessed using one-way ANOVA and Mann–Whitney U tests, while ureteral damage was evaluated using Chi-square tests. A p-value of 0.05 or less was considered statistically significant.

## Results

AR at C lasing modes (0.5 J/10 Hz and 0.5 J/20 Hz) showed no significant differences between trainees and seniors (p: 0.33 and p: 0.54, respectively). Comparison of these two frequencies also revealed similar ablation rates in both expertise groups (p: 0.25) (Table 2). B mode showed similar AR across different settings with no significant difference between them (p: 0.74), nor between levels of expertise (p: 0.92) (Table 2). When comparing overall AR between C and B lasing modes, no significant differences were observed (p: 0.96). The highest ablation rate, 85.71%, was achieved by a senior using the 0.5 J/20 Hz continuous laser setting after delivering 1.5 kJ of energy.

**Table 2:** Ablation rate

	Laser settings	Ablation rate (Δmg/mg, $\overline{x}$ ± S.D.)			
		Residents (n = 5)	Senior (n = 5)	*p* value	
Continuous	0.5 J/10 Hz	56.55 ± 7.11	70.08 ± 15.72	0.246	0.964
	0.5 J/20 Hz	67.44 ± 13.23	64.55 ± 22.23		
	Total	62.00 ± 11.21	67.31 ± 18.38	0.471	
Burst	0.5 J/20 Hz	68.73 ± 17.3	56.17 ± 13.99	0.742	
	0.5 J/30 Hz	77.15 ± 8.18	70.91 ± 11.59		
	0.5 J/60 Hz	62.52 ± 20.78	68.58 ± 12.36		
	0.1 J/200 Hz	48.64 ± 29.27	67.88 ± 10.07		
	0.05 J/400 Hz	57.56 ± 11.0	64.03 ± 14.96		
	Total	62.92 ± 19.48	65.51 ± 12.47	0.916	

These ablation rates represent the effectiveness of the laser settings in the study. The data is categorized by different laser energy settings by continuous mode and burst, with separate results for residents and seniors. Notably, all tests maintained a consistent total energy delivery of 1.5 kJ

Ureteral injuries were assessed on a damage scale ranging from 1 to 3. Notably, none burn-lesions (type 3) were found in the C mode. At this lasing mode, there were no significant differences in thermal injury between laser settings (p: 0.71) or between levels of expertise (p: 0.97) (Table 3). For B mode, a variety of lesions, including up to 11 cases of type 3 lesions, were observed (Table 3). Specifically, at 0.5 J/20 Hz, there were more type 3 lesions in the resident's group (p: 0.04). However, when comparing all B lasing settings per expertise, no significant differences were found (p: 0.11). Additionally, when comparing between laser settings, no significant differences were observed (p: 0.83). A secondary analysis comparing low-frequency and high-frequency settings (0.5 J/20 Hz vs. the others) found no differences (p: 0.48), nor with 0.5 J/20 Hz and 0.5 J/30 Hz vs. the others (p: 0.17).

**Table 3:** Thermal injury from laser lithotripsy (scale 1–3)

	Laser settings	Damage scale	n (%)		p value	
			Residents	Senior		
Continuous	0.5 J/10 Hz	1	4 (80%)	4 (80%)	0.709	0.048*
		2	1 (20%)	1 (20%)		
		3	0	0		
	0.5 J/20 Hz	1	3 (60%)	5 (100%)		
		2	2 (40%)	0		
		3	0	0		
	Total	1	7 (70%)	9 (90%)	0.97	
		2	3 (30%)	1 (10%)		
		3	0	0		
Burst	0.5 J/20 Hz	1	1 (20%)	5 (100%)	0.827	
		2	1 (20%)	0		
		3	3 (60%)	0		
	0.5 J/30 Hz	1	2 (40%)	5 (100%		
		2	1 (20%)	0		
		3	2 (40%)	0		
	0.5 J/60 Hz	1	2 (40%)	2 (40%)		
		2	2 (40%)	2 (40%)		
		3	1 (20%)	1 (20%)		
	0.1 J/200 Hz	1	2 (40%)	3 (60%)		
		2	2 (40%)	1 (20%)		
		3	1 (20%)	1 (20%)		
	0.05 J/400 Hz	1	3 (60%)	2 (40%)		
		2	1 (20%)	2 (40%)		
		3	1 (20%)	1 (20%)		
	Total	1	10 (40%)	17 (68%)	0.109	
		2	7 (28%)	5 (20%)		
		3	8 (32%)	3 (12%)		

Thermal injury resulting from different laser settings and modes being rated on a damage scale from 1 to 3. The table provides a breakdown of the cases of observed injuries (n) and the corresponding percentages (%)

*Comparison between overall continuous setting tests versus burst settings tests (p value < 0.05)

For all tests, no significant differences in ureteral damage were found between levels of expertise (p: 0.76). However, among both lasing techniques, higher ureteral damage lesions were observed in the B group: type 3 lesions (n = 11 in the B group vs. n = 0 in the C group) (p: 0.048) (Table 3).

## Discussion

To our knowledge, this is the first study comparing these two lasing techniques, revealing comparable stone ablation with equal energy delivery. The B group showed better efficacy, but higher thermal lesions incidence compared to the C mode. The lower incidence of direct thermal lesions provides practical guidance for urologists, suggesting that a C lasing mode is safer than the B mode. It is crucial to consider both efficacy and safety parameters when tailoring laser settings for endoscopic procedures.

Since the development of high-power lasers, endourologists and laser companies have pushed to use higher frequency and higher power settings to improve stone-free rates in less time, but show no significant benefits over lower settings, as per recent meta-analyses [19–22]. The absence of significant differences in AR between C and B lasing modes suggests that both techniques can achieve comparable stone fragmentation. This consistency holds across different frequencies and energy settings tested in our study.

On the other hand, in terms of safety, it is well known that increasing power proportionally increases fluid temperature, causing indirect thermal damage due to protein denaturation, which may lead to devastating complications like ureteral stricture formation. Additionally, thermal injury burns after direct contact of the laser emission have also been described at high pulse rate frequency settings [13, 23, 24]. The disparity in ureteral damage in the present study, with a higher incidence of type 3 lesions in the B group and higher frequency setting group, emphasizes the importance of careful consideration when selecting lasing techniques.

The outcomes stratified by levels of expertise revealed no significant differences in stone ablation rates or ureteral damage between trainees and seniors. This suggests that the observed advantages of the continuous mode, particularly at 20 Hz, are applicable across different skill levels. The consistent performance across expertise levels strengthens the generalizability of our findings.

While our study provides valuable insights into the safety and efficacy of different lasing techniques, it is essential to acknowledge certain limitations. Limitations include the in vitro setup's inability to fully mimic in vivo conditions, though efforts were made to replicate real-world irrigation practices. [24, 25]. Moreover, in terms of safety, there are two types of thermal injuries: indirect damage caused by the increase of temperature of the irrigation fluid and, as in this study, due to direct contact with the laser and the mucosa. Even though we didn’t measure the temperature during the trial, we maintained a constant irrigation. Therefore, the impact of high-temperature irrigation fluid was negligible. As detailed by Aldoukhi et al., with power up to 40 W (which exceeds our settings), only a maximum of 38.5 °C was achieved with proper irrigation [25, 26]. Moreover, increasing power proportionally increases fluid temperature, irrespective of the ratio between energy and frequency and potentially injurious temperatures were reached with power up to 40 W (we used a max of 30 W) [27, 28]. So thermal impacts shown in ureteral models are almost certainly a result of direct laser irradiation of the mock ureteral wall and not due an increase of the temperature in the irrigation medium. Additionally, sample size and use of stone phantoms instead of human stones may limit generalizability, and alternative burst settings could be explored. Notably, the burst lasing technique is associated with a higher range of total average power, introducing a potential source of bias. However, it is crucial to acknowledge that the choice of the burst technique was intentional and aligns with the specific settings implemented across the experimental groups. In addition, the need to press and stop over the laser pedal may led to vibration of the scope in the hand of operators, which will happen in clinical practice. On the other hand, one of the limitations of our study pertains to the scale used for assessing ureteral injury. We acknowledge that the scale was specifically established for this publication based on findings from the surface. While this approach was undertaken to facilitate clear and comprehensive presentation of our findings, it is important to note that the scale lacks recognition and validation beyond the confines of our trial. Although efforts were made to ensure consistency through blinded classification by consensus between two authors, the subjectivity inherent in this process remains a concern. Additionally, our meticulous approach to data collection and analysis enhances the reliability and validity of our findings within the scope of this study.

## Conclusion

Regarding efficacy, C and B lasing techniques with TFL seems to achieve comparable stone ablation rates. However, in terms of safety, C mode at a maximum of 20 Hz causes less direct thermal lesions in the surrounding tissue than B lasering technique. These results provide valuable insights for urologists, especially when selecting appropriate laser settings for various clinical scenarios.

## Data Availability

Data repositories are available.
